# An Unusual Fat-Containing Presacral Tumor in an Elderly Patient

**DOI:** 10.1155/2014/674365

**Published:** 2014-02-06

**Authors:** Maria Inês Leite, Afonso Gonçalves, Ana Cristina Ferreira, Santiago Ortiz, Rui Esteves, Isabel Távora

**Affiliations:** ^1^Radiology Department, Hospital Santa Maria, 1649-035 Lisbon, Portugal; ^2^Pathology Department, Hospital Santa Maria, 1649-035 Lisbon, Portugal; ^3^Surgery Department, Hospital Santa Maria, 1649-035 Lisbon, Portugal

## Abstract

The authors present a case of a presacral myelolipoma diagnosed in an 84-year-old male patient with longstanding pelvic pain and past medical history of bladder cancer. Pelvic computed tomography (CT) revealed a well-encapsulated and lobulated presacral mass, with mixed fat and soft-tissue attenuation. Magnetic resonance (MR) imaging provided further confirmation of macroscopic intralesional fat and excluded either adjacent bone invasion or bladder cancer recurrence. A presacral myelolipoma was suspected based on imaging findings, with liposarcoma and teratoma having also been considered for the differential diagnosis. The histological confirmation of the tumor was only attained postoperatively. This case report alerts to the possible presacral location of myelolipomas, which should be considered for every fat-containing lesion detected in this region. The main clinical, imaging, and differential diagnoses of this entity are reviewed in this paper.

## 1. Introduction

Myelolipomas are benign tumors composed of both mature adipose and hematopoietic elements, most commonly occurring in the adrenal glands [1, 2].

This case report alerts to the possibility of presacral myelolipomas, which have been only occasionally reported in the literature but should nevertheless be considered in the differential diagnosis of every fat-containing tumor occurring in this region [3–10].

The imaging findings of these tumors completely overlap with those of their adrenal counterpart, as was observed in this case. Notwithstanding, other possible presacral tumors, such as teratomas and liposarcomas, may have similar CT and MRI appearance and therefore the definite diagnosis still requires histological confirmation [8–12].

## 2. Case Report

An 84-year-old male patient was admitted to our hospital with complaints of longstanding pelvic pain with more than six months of duration and significant worsening during the past month. He denied changes in bowel habits and urinary symptoms. The patient had a medical history of bladder cancer treated conservatively. Other medical problems included arterial hypertension and benign prostatic hypertrophy.

**Figure 1:**
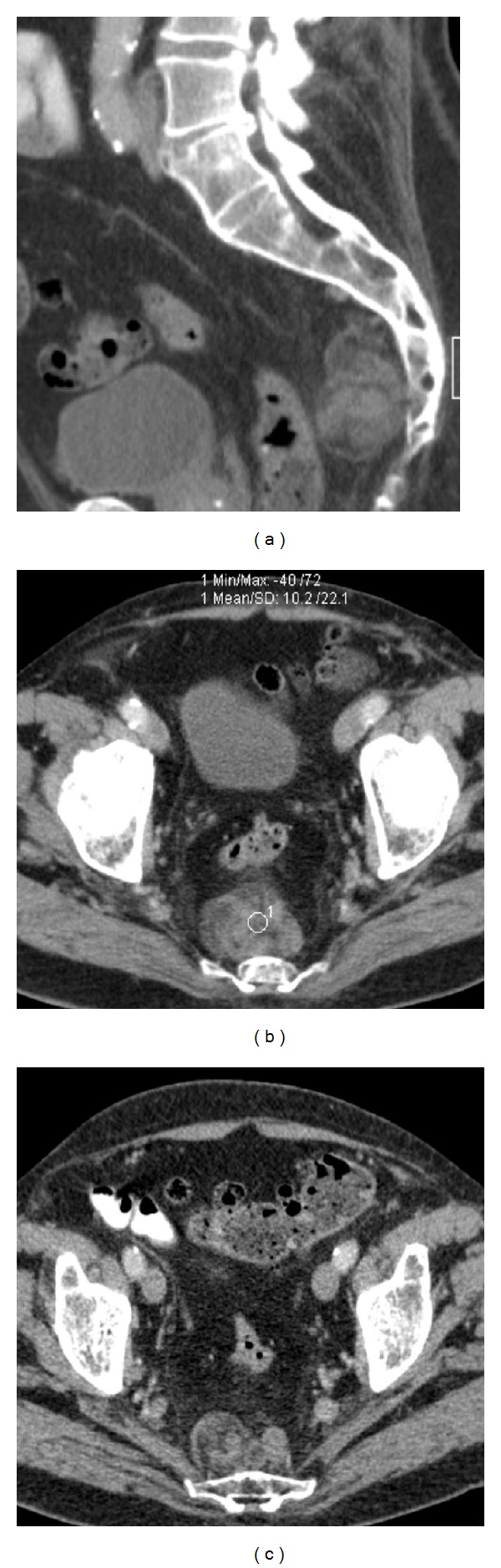
84-year-old male with presacral myelolipoma. Sagittal (a) and axial ((b), (c)) contrast-enhanced CT images show the presacral mass, spanning from S3 to S5. It presented lobulated contours and a heterogeneous appearance, due to an admixture of soft-tissue and fat elements. The latter had attenuation values of about −40 HU. The mass was located in the retrorectal space and adhered to the sacrum, posteriorly.

Pelvic contrast-enhanced CT revealed a well-encapsulated and lobulated solid mass posterior to the rectosigmoid colon, measuring 5 cm in greatest dimension. It abutted the sacrum posteriorly and was located between S3 and S5 (Figure 1(a)). The mass presented a heterogeneous appearance due to a mixed fat (attenuation values as low as −40 HU) and soft-tissue composition and had no calcifications (Figures 1(a)–1(c)).

**Figure 2:**
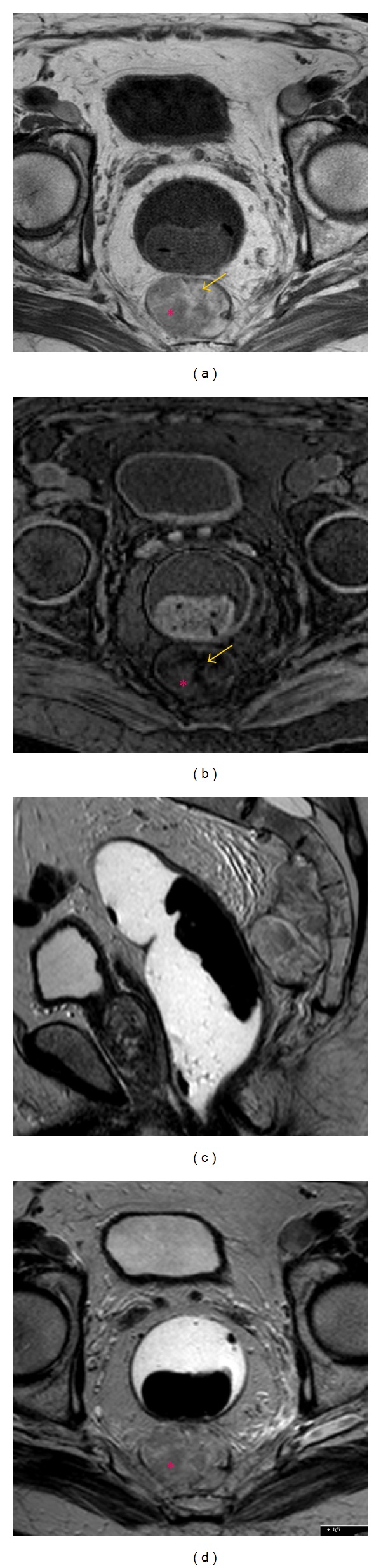
84-year-old male with presacral myelolipoma. Precontrast T1-weighted (a) and fat-suppressed T1-weighted (b) FSE axial images depicted the presence of macroscopic fat (arrows). The solid hematopoietic elements (asterisk) exhibited intermediate signal on precontrast T2-weighted sagittal (c) and axial (d) images and low-signal intensity on T1-weighted sequences.

**Figure 3:**
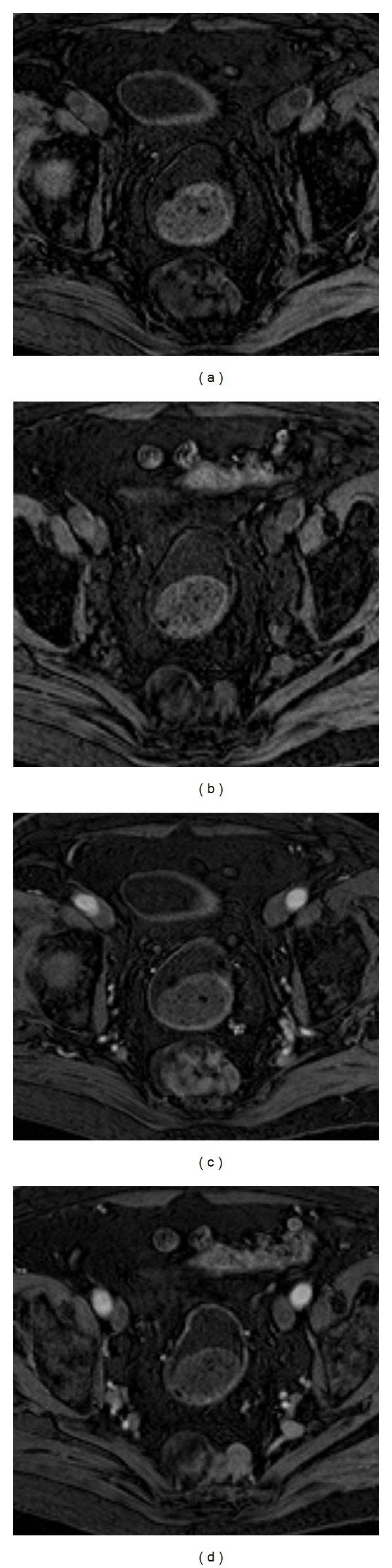
84-year-old male with presacral myelolipoma. Postcontrast T1-weighted fat-suppressed axial images at a higher (a) and lower (b) level of the mass demonstrated avid gadolinium uptake of the solid hematopoietic components of the lesion.

Subsequent pelvic MR imaging was performed in order to provide further characterization of the tumor. The presence of macroscopic fat was confirmed by the presence of components with high signal intensity on T1-weighted sequences that demonstrated a drop of signal on corresponding fat-suppressed T1-weighted sequences (Figures 2(a) and 2(b)). The soft-tissue component exhibited low and intermediate signal intensity on T1- and T2-weighted sequences (Figures 2(a)–2(d)), respectively, and avid gadolinium uptake (Figures 3(a) and 3(b)). Posterior bowel wall invasion was excluded by the evidence of a thin but intact intervening fat plane between the tumor and the muscular layer of the rectosigmoid colon. There were no signs of adjacent sacral bone or sacral nerve roots invasion, which was better demonstrated on sagittal T2-weighted sequences (Figures 2(c) and 2(d)). The bladder wall was diffusely thickened suggesting chronic outflow obstruction related to benign prostatic hypertrophy (Figures 1(a) and 2(c)). Pelvic lymphadenopathies were not detected.

Based on the imaging findings, myelolipoma, liposarcoma, and teratoma were considered in the differential diagnosis.

**Figure 4:**
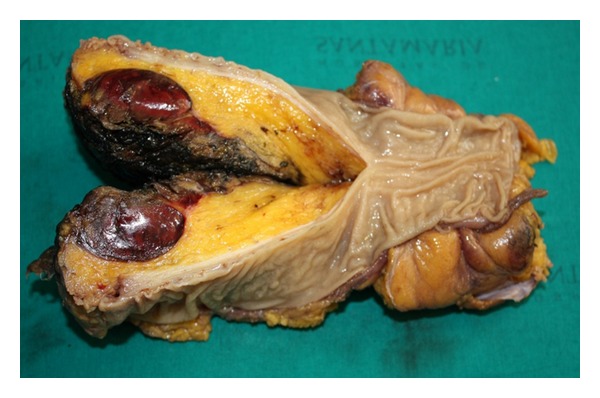
84-year-old male with presacral myelolipoma. Gross examination revealed a well-circumscribed, fleshy, and red-brown tumor, measuring 5,5 cm of greatest dimension. It was located in perirectal tissue, adjacent to the circumferential margin, and was 1 cm distant of the muscularis propria.

**Figure 5:**
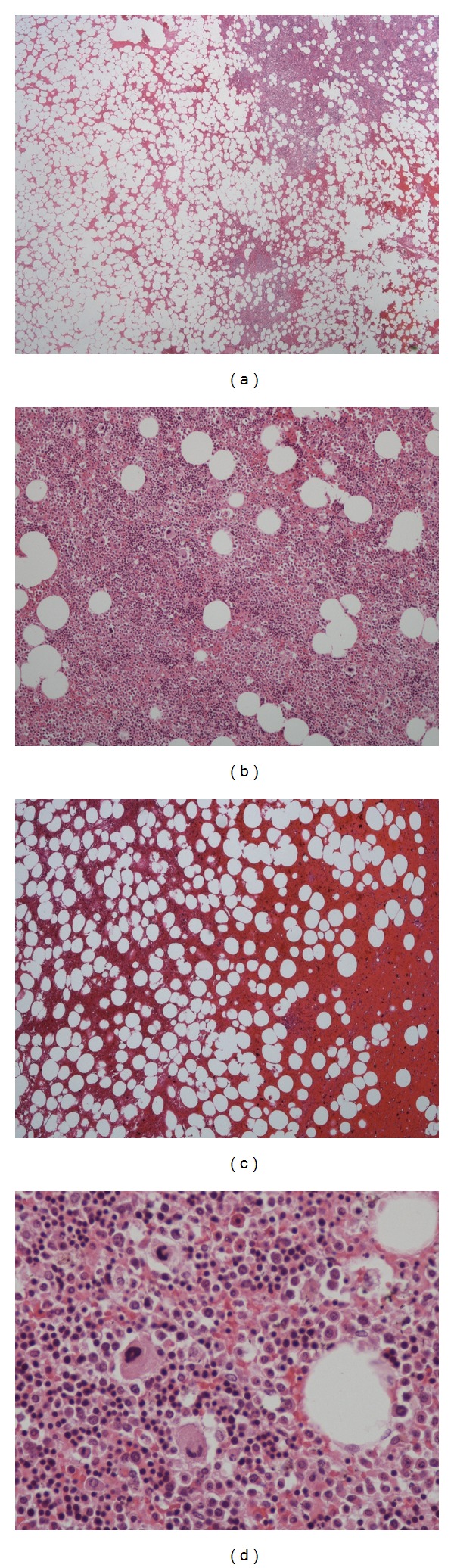
84-year-old male with presacral myelolipoma. Microscopic examination: the tumor was composed of varying amounts of mature adipose tissue admixed with hematopoietic elements ((a) and (b): H&E, x40); there were also dispersed hemorrhagic areas ((c): H&E, ×40). In higher magnification view the presence of the three cell lineages (myeloid, erythroid, and megakaryocytic) was evident, similarly to the normal bone marrow ((d): H&E, ×400).

Since the patient was symptomatic and surgery could not be precluded, a preoperative biopsy was not performed. The patient was submitted to a concurrent proctectomy (Hartmann type procedure) due to the evidence of extensive peri-rectal tissue involvement. On gross examination, a tumor with 5,5 × 4 × 3 cm of greatest dimensions was found in the perirectal adipose tissue (Figure 4). Histological examination of the resected specimen confirmed the diagnosis of myelolipoma (Figure 5).

The postoperative course was uneventful and after two years of follow-up there has been no evidence of local recurrence.

## 3. Discussion

Extra-adrenal myelolipomas are benign nonfunctioning tumors composed of mature adipose tissue and trilineage hematopoietic cells (megakaryocytic, myeloid, and erythroid cells). They are histologically indistinguishable from their adrenal counterpart and resemble the bone marrow tissue [1, 2].

To our knowledge, less than 50 cases of extra-adrenal myelolipomas have been published. Half of these have been reported in the presacral region, which constitutes their second most common location, only surpassed by an adrenal origin. In a minority of cases, perirenal, mediastinum, liver, and stomach locations have been reported in a minority of cases [3].

The etiology of extra-adrenal myelolipomas is still unknown, though it has been suggested that they might arise from reactivation of peritoneal connective tissue hematopoiesis or embolization of bone marrow tissue [3–5].

Presacral myelolipomas are usually diagnosed in elderly patients, usually between the fifth and seventh decades, as was the case of this patient. A 2 : 1 female to male predominance has been suggested [6, 7]. The presence of symptoms is directly related to the dimensions of the tumor. Although the majority of them are small and thus found incidentally, larger lesions do occur and a tumour measuring 26 cm has been reported in the literature [8]. They may present with symptoms, either due to intratumoral hemorrhage or compression. Patients may complain of urinary retention, constipation, or lower extremity radiculopathy/sciatica [6, 7, 9]. An association with diabetes, Cushing syndrome, Addison disease, adrenal hyperplasia, and chronic steroids intake has been reported [4, 6].

As was observed in this patient, presacral myelolipomas are typically round or oval well-circumscribed lesions that may adhere to the sacrum by direct contiguity. However, they do not invade the bone [1, 2, 9].

Identification of macroscopic intralesional fat is a fundamental milestone in the imaging diagnosis of myelolipomas. Even so, their imaging appearance can be quite diverse, depending on the relative proportion of fatty and hematopoietic elements [3, 9]. It has been suggested that extra-adrenal lesions contain less intralesional fat than those of adrenal origin (50% versus 90%) [5].

On ultrasonography, these tumors present a heterogeneous echogenicity and may range from hyperechoic to hypoechoic masses, according to the predominance of fat or hematopoietic elements, respectively [10]. On CT imaging, myelolipomas present as encapsulated masses with macroscopic fatty tissue interspersed with avidly enhancing hematopoietic soft tissue elements [3, 9]. The attenuation value of fat in myelolipomas may be lower than −20 HU, but it is usually higher than that of retroperitoneal fat because of the admixture with hematopoietic tissue [5]. Intratumoral calcifications can be found in up to 10% of cases, as a result of previous hemorrhagic episodes [3, 5, 9]. MR imaging can confidently depict the presence of macroscopic fatty elements, which yield high signal intensity on T1-weighted images and demonstrate loss of signal on fat-suppressed T1-weighted sequences [3, 11]. In-phase and out-of-phase gradient recalled echo (GRE) sequences may be also helpful in differentiating microscopic from macroscopic fat, since the latter, typically observed in association with myelolipomas, does not lose signal on out-of-phase images [6]. Hematopoietic elements usually exhibit low-signal intensity on T1-weighted sequences and intermediate signal intensity on T2-weighted sequences. Technetium 99m (99mTc) sulfur colloid scintigraphy may be used to identify erythroid elements [5].

The differential diagnosis of presacral myelolipomas mainly includes other retroperitoneal tumors with fatty components, such as liposarcomas or teratomas, as well as extramedullary hematopoiesis. In addition, other non-fat-containing lesions commonly occurring in this region must be ruled out [3, 9].

Liposarcomas represent the most prevalent and aggressive fat-containing retroperitoneal tumors. One must be aware that lipomas seldom occur in the retroperitoneum and therefore the diagnosis must be approached with extreme caution, since many of them are misclassified due to biopsy sampling errors [12]. In terms of imaging, liposarcomas present as nonencapsulated masses with a predominant fat component and thick irregular or nodular septa. They usually exhibit an infiltrative growth pattern and occasionally extend to the sacral foramina [5, 9]. The presence of enhancing solid components, calcifications, or ossifications suggests dedifferentiation and a poorer prognosis [5, 12].

Teratomas may occasionally occur in the presacral region. However, their incidence is very low in older patients; a bimodal age distribution has been reported, one in the first six months of life and one in young adults [12]. Furthermore, the appearance of teratomas is suggested by the presence of both cystic and solid components, the former generally corresponding to mature tissue (either sebaceous material or hair). The concomitant presence of fat and calcifications is observed in 83% and 93% of cases, respectively [12].

Extramedullary hematopoiesis can be distinguished of myelolipomas based on the distribution, location, and morphology of the lesions. Their involvement is most frequently multifocal and predominantly affects the mediastinum; these lesions are usually poorly circumscribed and present minimal macroscopic fat. Moreover, an association with hepatosplenomegaly and chronic anemia is generally observed [3, 8, 13].

Neurofibromas and chordomas are usually easily distinguished from myelolipomas, since these do not usually contain fat components. Chordomas typically induce pain and neurologic defects due to extensive bone destruction, in opposition to neurofibromas, which cause only slight bone remodeling and may extend into the sacral foramina [6, 9].

The definitive diagnosis of presacral myelolipomas is still based on histology, given the potential overlapping imaging appearance with some of the more menacing fat-containing tumors described above [9, 13].

Given its benign nature, conservative management is indicated in asymptomatic nonhemorrhagic myelolipomas. Nonetheless, a pathological proven diagnosis obtained from percutaneous fine needle aspiration (FNA) and radiologic-pathologic concordance are required in such cases.

Surgery remains the treatment of choice for symptomatic myelolipomas, as was the case of our patient. Surgery is also the preferred approach in indeterminate cases [4, 8] and in large tumors (over 10 cm in size), the latter due to a potential risk of spontaneous hemorrhage and life-threatening shock [14].
